# The prospects of targeting messenger RNA surveillance factor PELOTA for developing virus resistance in plants

**DOI:** 10.1007/s11103-026-01700-2

**Published:** 2026-03-18

**Authors:** Anik Majumdar, Yeluru Mohan Babu, Firoz Mondal, Mehulee Sarkar, Prantik Mazumder, Priyanka Pradhan, Ravinder Kumar, Sota Koeda, Anirban Roy

**Affiliations:** 1 Advanced Centre for Plant Virology, Division of Plant Pathology, ICAR- Indian Agricultural Research Institute, Pusa, New Delhi, 110012 India; 2 Division of Crop Protection, ICAR-Indian Institute of Horticultural Research, Bengaluru, 560089 India; 3 Faculty of Agriculture, Kindai University, Nara, 631-8505 Nara Japan

**Keywords:** PELOTA, mRNA surveillance, Plant viruses, RNA quality control, Susceptibility, Resistance

## Abstract

The mRNA-surveillance factor, PELOTA is essential for maintaining translation fidelity and facilitating the degradation of faulty mRNAs. Initially recognized for its role in ribosome rescue and RNA quality control in eukaryotic cells, PELOTA has recently garnered attention for its diverse functions in plant biology as well as in plant-virus interactions. Numerous studies suggest that the PELOTA protein plays a distinctive role in determining either susceptibility or resistance to various plant pathogens, including viruses. Interestingly, PELOTA exhibits a dual function: while certain allelic variants confer resistance or susceptibility to diverse plant viruses, complete or severe disruption of its activity impairs fundamental processes of plant growth and development. In this review, we synthesize current knowledge of PELOTA-mediated mRNA surveillance across eukaryotes, critically examine its emerging role in shaping plant–virus dynamics, and assess the deleterious developmental consequences of its mutation in plants. Although disruption of PELOTA can enhance resistance to various pathogens, including viruses, it may also compromise RNA quality control and normal plant development. Thus, a thorough evaluation is essential before targeting PELOTA for engineering durable disease resistance in crops.

## Key Message

PELOTA maintains translational fidelity and ribosomal rescue. Although PELOTA manipulation can confer viral resistance, its essential role in mRNA surveillance and plant development necessitates careful evaluation for crop engineering.

## Introduction

Plant viruses pose a significant threat to global agriculture, leading to substantial crop losses and jeopardizing food security. To combat viral infections, plants have evolved various defense mechanisms, including RNA silencing, RNA degradation, and translation-coupled RNA quality control (RQC). These key RNA-mediated pathways are essential for targeting and degrading viral RNAs (Li and Wang 2019; Majumdar 2024). Notably, among the viruses inflicting considerable damage on crops, begomoviruses (family: *Geminiviridae*) are particularly prevalent (Piedra-Aguilera et al. 2019). A destructive begomovirus, *Begomovirus coheni* (commonly known as tomato yellow leaf curl virus, TYLCV), is the causative agent of the severe tomato yellow leaf curl disease (TYLCD) in tomato plants. Although various strategies have been explored for the management of TYLCD, cultivating resistant varieties has emerged as the most effective method for mitigating crop loss. To date, six resistance genes (*Ty-1* to *Ty-6*) have been identified from different wild tomato species, thereby facilitating the development of TYLCD-resistant cultivated tomato varieties (Yan et al. 2021; Mori et al. 2021). The tomato homolog of the messenger RNA surveillance factor PELOTA (encoded by the *Pelo* gene), which is involved in the ribosome recycling phase of protein synthesis, has been associated with the *ty-5* locus (Anbinder et al. 2009). The *ty-5* locus was identified in the tomato breeding line TY172, derived from four *Solanum peruvianum* accessions (PI126926, PI126930, PI390681, and LA0441) (Friedmann et al. 1998; Hutton et al. 2012; Lapidot et al. 2015) (From hereon, the wild-type allele of the *Pelo* gene, which confers susceptibility is designated as *Pelota*, mutant allele which confers resistance as *pelota*, and the protein encoded by them as PELOTA protein). Similarly, in another study, the *ty-5* locus containing the *pelota* was mapped in TYLCV-resistant tomato line AVTO1227 by a whole genome resequencing approach (Wang et al. 2018). In TYLCV-resistant tomato line TY172 (harboring *pelota*), the recessive locus *ty-5* was mapped to a 425-base pair region containing two genetic alterations: a transversion in the first exon (T^47^ to G) of the *Pelo* gene encoding PELOTA protein and another in its proximal promoter. Interestingly, the transcript levels of the gene remained unchanged regardless of TYLCV infection, indicating that resistance is determined by the polymorphism in the coding region, specifically the T^47^ to G transversion in the first exon of *Pelo*. This T^47^ to G transversion in the first exon of *Pelo* resulted in a Valine^16^-to-Glycine substitution in TY172. Additionally, through functional studies, it was found that silencing the *Pelo* gene in a susceptible tomato line M-82 conferred resistance to TYLCV while overexpressing the susceptible allele of the gene in the resistant line (TY172) rendered it susceptible. Conversely, overexpressing the resistant allele (*pelota*) in susceptible plants did not alter their susceptibility. These findings confirmed that *Pelo* is the gene responsible for resistance at the *ty-5* locus.

In addition to its mRNA surveillance activity, PELOTA protein is highly conserved among all the eukaryotic organisms and has a significant role in plant growth and development (Kong et al. 2021; Ding et al. 2018; Guo and Gregory 2023). Recently, a review by Li et al. (2025) has highlighted the dual role of *Pelo* gene in conferring both resistance and susceptibility to different RNA and DNA plant viruses. Unlike the previous article, which primarily focuses on the dual roles of *Pelo* gene in conferring plant susceptibility and resistance to viruses, our review provides a uniquely comprehensive perspective by integrating the evolutionary conservation and mechanistic basis of PELOTA’s function in mRNA surveillance across plants, mammals, and yeasts. This article thoroughly details how PELOTA protein and its partners orchestrate ribosome rescue and translational quality control through pathways such as no-go decay (NGD) and nonstop decay (NSD). Furthermore, this review explicitly emphasizes the significant negative impact of *Pelo* mutations on plant growth and development, including disruptions in developmental pathways. Finally, by bridging these insights, this article provides a more mechanistic and comparative context for understanding how PELOTA protein-mediated mRNA surveillance intersects with plant-virus interactions, offering a deeper and broader synthesis than previous literature.

## PELOTA protein and its function in mRNA surveillance across different eukaryotes

**Fig. 1:**
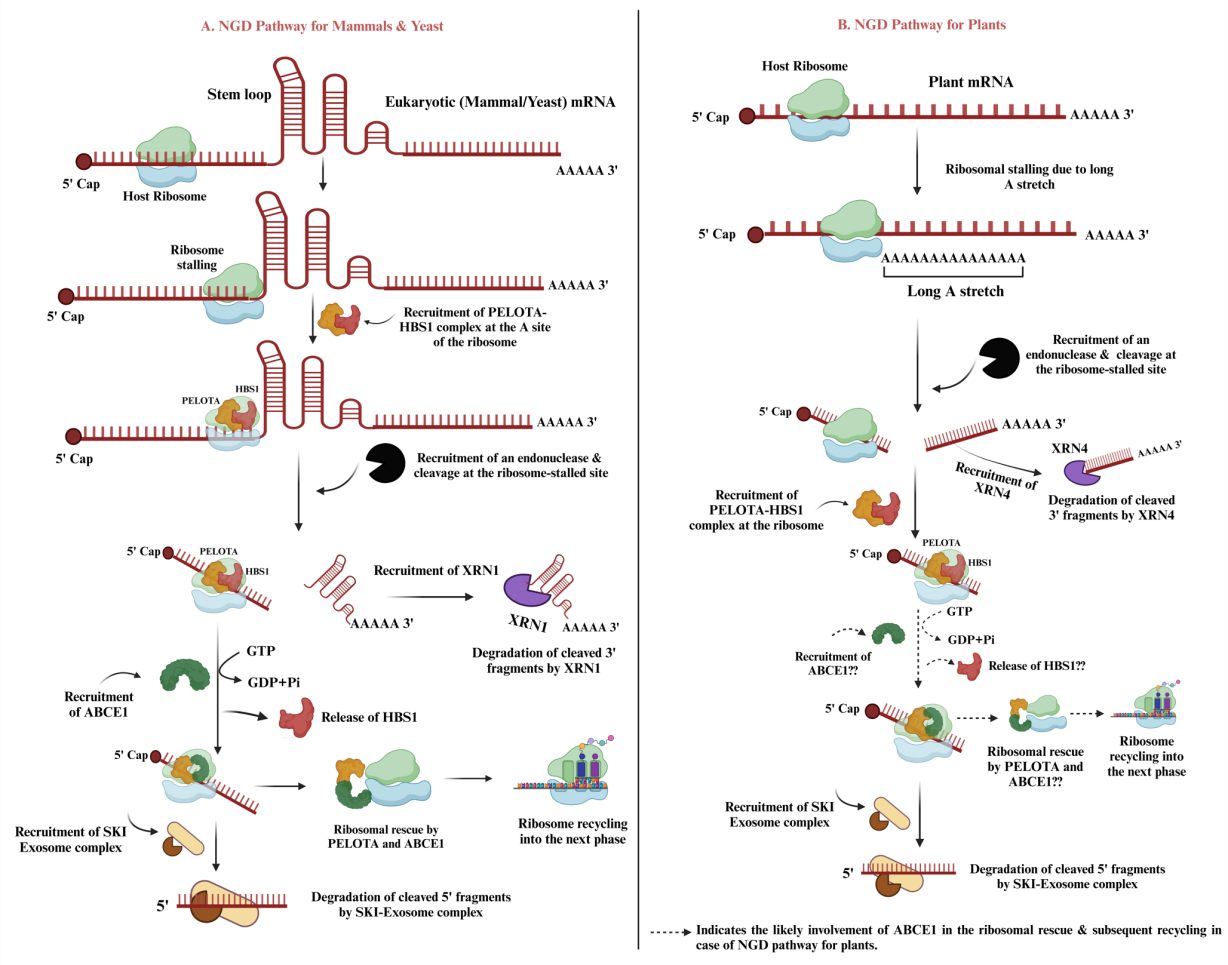
PELOTA-HBS1 mediated degradation of aberrant mRNA through NGD pathway in mammals, yeast, and plants: **A** In mammals and yeasts, during translation, when prolonged ribosomal stalling occurs due to loop-like/secondary mRNA structures, the PELOTA-HBS1 complex recognizes stalled ribosomes. As a result, an endonuclease, which cleaves such mRNA, is recruited at the ribosome stalled site. Following the cleavage, ABCE1 is recruited, and along with PELOTA, it rescues the stalled ribosome. XRN1 exonucleases and the cytoplasmic SKI-exosome complex further degrade the cleaved mRNA fragments. **B** In plants, NGD is triggered exclusively when long A-stretches appear within the mRNA coding region. This activates an unidentified endonuclease that induces endonucleolytic cleavage at the ribosome stalled sites. After the mRNA cleavage, the PELOTA-HBS1 complex binds to the stalled ribosome. The HBS1 is probably released in the subsequent steps, allowing ABCE1 and PELOTA to rescue the stalled ribosome. Consequently, the resulting 5' and 3' cleaved mRNA fragments are further degraded by the SKI-exosome complex and XRN4, respectively. This diagram of the plant NGD pathway is inspired by the previous study (Szádéczky-Kardoss et al. 2018b). This figure was created using Biorender

In eukaryotic cells, RNA quality control (RQC) systems play a crucial role in identifying and eliminating defective mRNAs, thereby preventing the accumulation of toxic or malfunctioning proteins. The role of PELOTA protein in RQC and other immune-related responses have been well established in recent years across mammals and yeasts **(**Table 1**)**. Currently, three primary mRNA surveillance mechanisms have been identified: nonsense-mediated mRNA decay (NMD) (Peltz et al. 1993), NSD, and NGD (Kong et al. 2021). NMD specifically targets mRNAs that contain premature termination codons (PTCs) and is activated when ribosomes encounter these codons during translation (Kervestin and Jacobson 2012). As the most extensively studied RQC pathway, NMD has been associated with anti-viral activity in both plants and animals (Li et al. 2019; Zhao et al. 2020). For example, NMD has been shown to limit viral accumulation in the case of pepino mosaic virus (PepMV) featuring m^6^A modifications, pea enation mosaic virus 2 with an unusual long 3’ untranslated region (UTR), and potato virus X (PVX) as well as turnip crinkle virus (TCV) that possess internally located termination codons (iTCs) (Garcia et al. 2014; He et al. 2024). Furthermore, PELOTA-mediated RQC has been reported to degrade RNA from potyviruses and help suppress infections caused by bamboo mosaic virus (Ge et al. 2023; Gandhi et al. 2008). Alongside NMD, NGD has emerged as a novel viral restriction pathway in plants (Ge et al. 2023). In yeasts and animals, the NGD pathway specifically targets mRNAs that harbour elongation-inhibiting structures during translation (Doma and Parker 2006; Becker et al. 2012; Pisareva et al. 2011) (Fig. 1A). However, in the case of plants, NGD is activated only when long A-stretches are found in the coding region of the mRNA (Fig. 1B). NGD activation also depends on the position of the A-stretch, with a longer distance from the start codon causing more efficient NGD induction in plants (Auth et al. 2021). At the ribosome-stalled site, an unknown endonuclease is activated and induces an endonucleolytic cleavage. The ribosome runs to the 3’ end of the cleaved fragment, followed by binding of the PELOTA- Hsp70 subfamily B Suppressor (HBS1) complex at the ribosome. In the consequent steps, it has been proposed that the HBS1 is released through GTP hydrolysis and ATP-Binding Cassette Sub-Family E Member 1 (ABCE1) is recruited. The PELOTA-ABCE1 complex rescues the ribosome, followed by degradation of the 5’ and 3’ cleaved fragments by the super killer (SKI)-Exosome complex and exoribonuclease 4 (XRN4) respectively (Szádéczky-Kardoss et al. 2018b). These translation-dependent quality control pathways facilitate the rapid degradation of defective transcripts, promoting ribosome dissociation and recycling to ensure error-free protein synthesis (Shoemaker et al. 2012). Additionally, both NSD and NGD are linked to protein quality control systems, such as the Ribosome-associated Quality Control (RaQC) system, which degrades proteins produced from faulty transcripts (Defenouillère and Fromont-Racine 2017). Beyond their role in removing defective transcripts, RQC systems also regulate the expression of normal wild-type mRNAs, underscoring their broader significance in maintaining cellular quality control and regulatory processes.

**Fig. 2:**
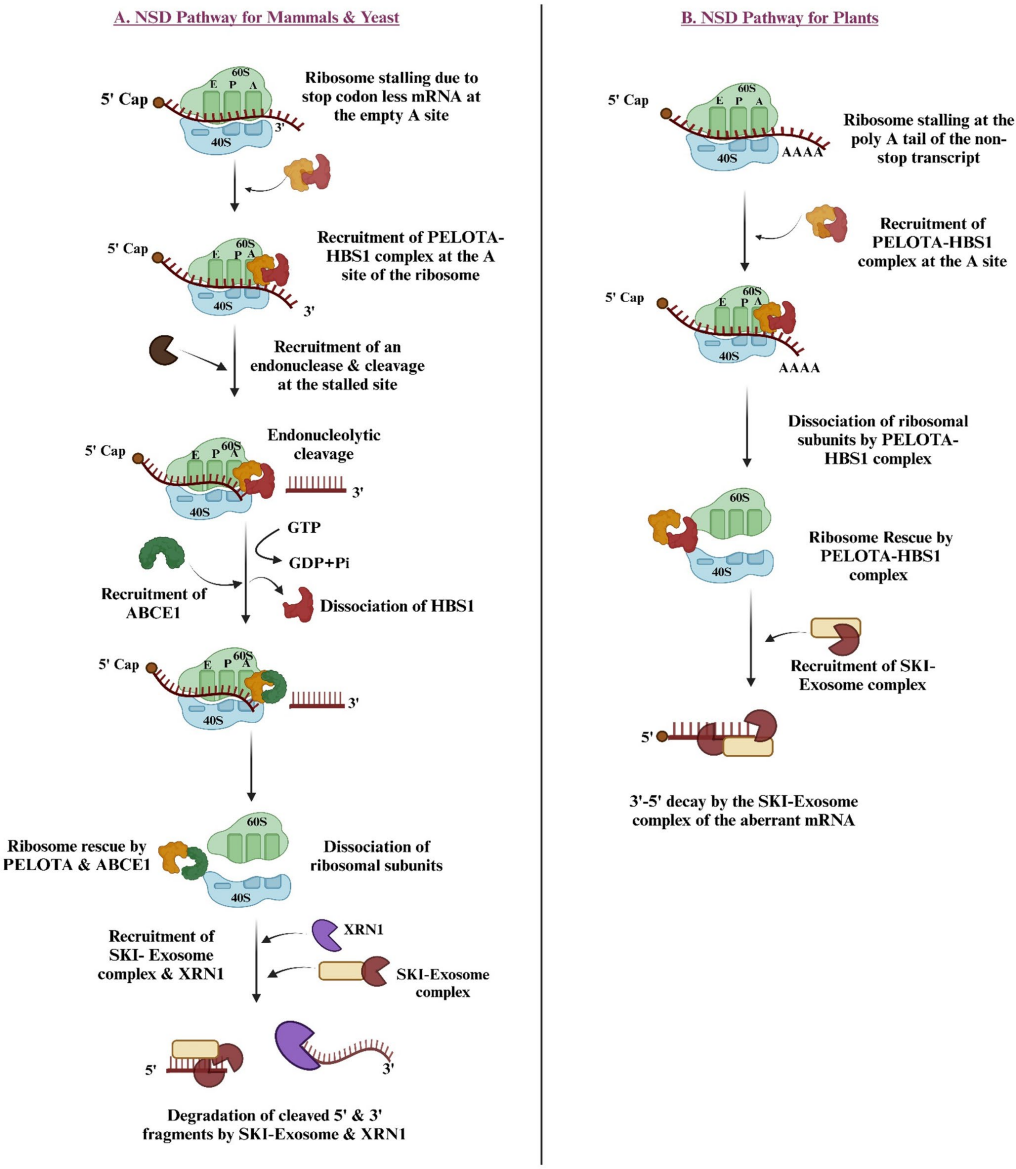
PELOTA-HBS1 mediated degradation of stop codon-less mRNA through NSD pathway in mammals, yeasts, and plants: **A** In mammals and yeast, ribosomal stalling due to stop codon-less mRNA is recognized by the PELOTA-HBS1 complex at the empty A site. HBS1 promotes the binding of PELOTA to the A-site of the 60 S subunit of the ribosome. An endonuclease is recruited, which cuts the mRNA at the stalled site. Following this, ABCE1 is recruited, and together with PELOTA it rescues the stalled ribosome. Further degradation of cleaved mRNA fragments occurs with XRN1 exonucleases and cytoplasmic exosomes. **B** The proposed NSD model for plants suggests that ribosome stalling takes place when it encounters the poly A tail of a nonstop mRNA. This leads to the recruitment of the PELOTA-HBS1 complex, which binds to the A-site of the stalled ribosome and facilitates the attachment of the SKI-Exosome complex. Consequently, the faulty mRNA is degraded by the SKI-Exosome complex, allowing the ribosome to be rescued by the PELOTA-HBS1 complex. This diagram of the plant NSD pathway is based on the model proposed by (Szádéczky-Kardoss et al. 2018a). This figure was created using Biorender.

NSD (nonstop decay) and NGD (no-go decay) are inter-related RNA quality control mechanisms that depend on the PELOTA protein (known as Dom34 in yeast) and HBS1 for their activity (Tsuboi et al. 2012). NSD specifically targets two types of mRNAs lacking stop codons: nonstop mRNAs with poly A sequences due to premature polyadenylation, and stop codon-less mRNAs, which are generated by endonucleolytic cleavage within the coding region. These defective mRNAs are recognized during the termination phase of translation (Frishmeyer et al. 2002). Under normal translation termination, the eukaryotic release factor 1 (eRF1)-eRF3-guanosine triphosphate (GTP) complex binds to the ribosome’s A-site when a stop codon is reached. GTP hydrolysis by eRF3 causes its dissociation and structural rearrangements in eRF1. Subsequently, ABCE1 is recruited, enabling the release of the synthesized peptide and ribosome recycling (Brandman and Hegde 2016; Jackson et al. 2012). However, in the case of mammals and yeasts, during ribosomal stalling due to stop codon-less mRNA, the PELOTA -HBS1 complex is recruited to the empty A-site of the ribosomes (Inada 2013) (Fig. 2A). HBS1 performs a role similar to eRF3, while PELOTA resembles eRF1 but lacks the sequences needed to recognize stop codons or release peptides. At the stalled site, an endonuclease is recruited, causing cleavage of the mRNA. GTP hydrolysis by HBS1 induces its dissociation and recruitment of ABCE1. PELOTA-ABCE1 complex rescues the stalled ribosome, and the 5’,3’ cleaved fragments are then rapidly degraded by the SKI-Exosome complex and XRN1 (Halbach et al. 2013; Nagarajan et al. 2013). Contradictory, the proposed NSD model for plants depicts that, ribosome stalling occurs when it runs into the poly-A tail of a nonstop mRNA (Fig. 2B). As a result, binding of the PELOTA -HBS1 complex occurs at the A-site of the stalled ribosome. The PELOTA-HBS1 complex dissociates the ribosomal subunits, followed by its rescue. Consequently, the faulty mRNA is degraded by the SKI-Exosome complex (Szádéczky-Kardoss et al. 2018a). Thus, in mammals, yeast, and plants, the PELOTA-HBS1 complex (Dom34–HBS1 in yeast) plays a key role in rescuing stalled ribosomes and initiating their recycling. This complex is essential for the function of NMD, NSD, and NGD pathways, which collectively ensure effective mRNA surveillance and protein quality control (Shoemaker et al. 2010; Hilal et al. 2016).

**Table 1:** Key literature on PELOTA/Dom34 function in RQC, and immune responses across different organisms

Type of organisms	Key findings	References
Yeasts	PELOTA-Dom34/HBS1 complex recognizes stalled ribosomes for mRNA decay, an essential process for clearing defective transcripts during stress-induced immune challenges.	Kobayashi et al. (2010)
Mammals	During mammalian translation, PELOTA-HBS1 induces dissociation of elongation complexes along with ABCE1, where PELOTA and ABCE1 were essential, but HBS1 had a stimulatory effect.	Pisareva et al. (2011)
Yeasts	The Dom34-HBS1 complex plays an essential role in the RNA quality control system by dissociating the stalled ribosome at the 3’ end of aberrant mRNA.	Ptsuboi et al. (2012)
Yeasts	The Dom34-HBS1 complex, along with RLi1 NTPase dissociates inactive ribosomes, thereby allowing translational restart in yeasts after glucose starvation.	Van Den Elzen et al. (2014)
Humans	The human PELOTA compensates for Dom34 functions in RNA quality control in yeasts, suggesting that their role in translational arrest is highly conserved.	Ikeuchi et al. (2016)
Bovine	Bovine (*Bos taurus*) bta-miR-2411 inhibits bovine viral diarrhea virus replication by suppressing PELOTA expression, suggesting a crucial role of PELOTA in facilitating viral replication.	Shi et al. (2018)
Mice	The paper highlights the non-canonical role of PELOTA in immune activation upon infection of mice with two bacterial pathogens, *Listeria monocytogenes* and *Salmonella enterica* serovar *Typhimurium*.	Magri and Poltorak (2024)
Mammals and *Caenorhabditis elegans*	Another non-canonical role of PELOTA highlights its role in mitigating premature senescence in cultured human cells, ageing of muscles in mice, and neuropathology in organoid and cellular models of Alzheimer’s disease.	Lee et al. (2025)

### PELOTA protein confers both susceptibility and resistance to plant viruses

In the complex world of plant-virus interactions, the *Pelo* gene plays an important role in susceptibility and resistance to plant viruses **(**Table 2**)**. As previously stated, PELOTA, a highly conserved eukaryotic protein, is well known for its involvement in ribosome rescue during stalled translation. However, recent studies have shown that its effect extends far beyond this function, affecting important pathways in the plant resistance/susceptible response to viruses. Interestingly, in most of the cases, the wild-type allele of *Pelo* gene has been found as a major susceptibility factor, capable of increasing viral replication and accumulation under certain conditions, while its absence or mutant form can confer resistance to plant viruses. These phenomena emphasize the critical significance of the *Pelo* gene in maintaining the delicate balance between host defense and viral survival in plants. The next sections will look at a few recent significant studies that demonstrate its distinct role in plant-virus interactions.

**Table 2:** The unique role of PELOTA across different plant-pathosystems, conferring either susceptibility/resistance to diverse plant viruses

Host Plant	Virus	Type of PELOTA mutation/alteration	Resulting phenotype	References
Rice	Southern rice black-streaked dwarf virus (SRBSDV)	Knockdown/knockout	Resistant	Sun et al. (2024)
Pepper	Pepper yellow leaf curl Indonesia virus (PepYLCIV) and pepper yellow leaf curl Aceh virus (PepYLCAV)	Recessive mutant allele of PELOTA, *pepy-1*	Resistant	Koeda et al. (2021)
*Nicotiana benthamiana*	Tomato yellow leaf curl China virus, tomato leaf curl Yunnan virus	Knockdown of the susceptible allele	Resistant	Ren et al. (2022)
Tomato	Tomato yellow leaf curl virus (TYLCV)	Overexpression of the susceptible allele in the resistant tomato line TY172	Susceptible	Lapidot et al. (2015)
Tomato	TYLCV	Silencing of the susceptible allele in susceptible tomato plants	Resistant	Lapidot et al. (2015)
Tomato	TYLCV	Knockout of the susceptible allele	Resistant	Pramanik et al. (2021)
Melon	TYLCV	Complementation of melon *Pelota* into a *ty-5* containing tomato line	Susceptible	Siskos et al. (2023)
*Arabidopsis thaliana* and *Nicotiana benthamiana*	Turnip mosaic virus (TuMV)	SUMOylated PELOTA-HBS1 complex	Resistant	Ge et al. (2023)
*Arabidopsis thaliana* and *Nicotiana benthamiana*	Turnip mosaic virus	PELOTA SUMOylation decoy by TuMV *NIb* encoded RNA-dependent RNA polymerase	Susceptible	Ge et al. (2025)
*Arabidopsis thaliana*	Beet curly top virus	Complementation of susceptible allele *Pepy-1* and resistant allele *pepy-1* from pepper into *A. thaliana Pelota1* knockout mutants	*Pepy-1* restored susceptibility, while *pepy-1* slightly compromised resistance in *A. thaliana Pelota1* knockout mutants	Onouchi et al. (2025)
Pepper	Mixed infection of pepper yellow leaf curl Indonesia virus (PepYLCIV) and pepper yellow leaf curl Aceh virus (PepYLCAV).	Recessive allele *pepy-1* and dominant allele *Pepy-2* (encodes RNA-dependent RNA polymerase).	Pyramiding *pepy-1* and *Pepy-2* in pepper confer resistance to PepYLCIV and PepYLCAV.	Koeda et al. (2025)

### PELOTA confers resistance to rice plants by inhibiting virus replication in both insect-vector and host plant, while also conferring susceptibility by viruses-mediated low accumulation

**Fig. 3:**
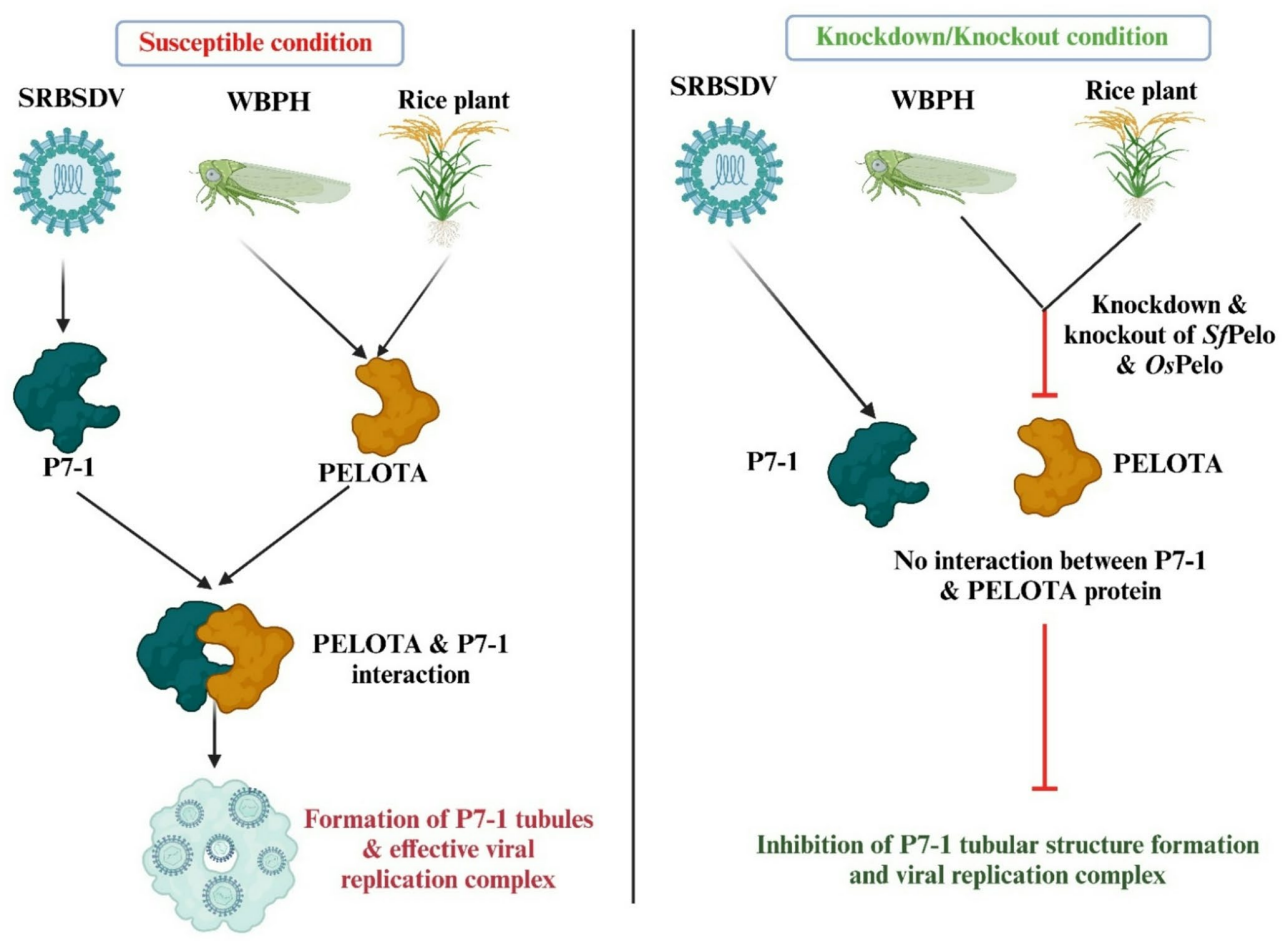
The role of PELOTA protein under susceptible and resistant conditions in Rice-SRBSDV-WBPH pathosystem: Under susceptible conditions, SRBSDV-encoded tubular protein P7-1 interacts with PELOTA protein in both rice and white-backed plant hopper, a key interaction required for the formation of viral replication complexes. However, knock-down and knock-out of *Pelo* expression in both WBPH and rice disrupted P7-1 tubule formation, thus inhibiting virus replication, and conferring resistance. This figure was created using Biorender.

Sun et al. (2024) demonstrated that *Pelo* gene plays a crucial role in limiting the spread of the southern rice black-streaked dwarf virus (SRBSDV) in transgenic rice plants and its insect vector, the white-backed planthopper (WBPH) (*Sogatella furcifera*). The study revealed that under susceptible conditions, the SRBSDV-encoded tubular protein P7-1 interacts with the PELOTA protein in both rice and planthoppers, a critical interaction necessary for the formation of tubules essential for viral replication (Fig. 3). However, alterations in *Pelota* allele expression in both rice and insect cells exhibited significant antiviral effects. In insect cells, either overexpression or knockdown of *Pelota* allele of *SfPelo* disrupted P7-1 tubule formation, consequently inhibiting viral activity. Similarly, knocking out *Pelota* allele expression of *OsPelo* in transgenic rice plants reduced the effective propagation of SRBSDV as well as two other rice viruses from different families. As a counter-strategy, the virus employs mechanisms to evade mRNA quality control systems, ensuring its persistence within the host. The study found that SRBSDV slightly reduces the accumulation of *OsPelo* in rice and *SfPelo* in *S*. *furcifera*, thereby undermining the mRNA surveillance mechanisms of its hosts. This slight reduction in *Pelo* expression levels prevents excessive inhibition of P7-1 tubule formation mediated by the PELOTA-HBS1 complex, thus facilitating the efficient propagation of the virus in both the plant and its insect vector.

### A recessive gene *pepy-1* encoding PELOTA confers resistance to begomoviruses in *Capsicum annuum*

Koeda et al. (2021) demonstrated that the mutant allele of the * Pelo* gene not only provides resistance in tomatoes but also in chili peppers (*Capsicum annuum*) against begomoviruses. They discovered a recessive gene, designated as *pepy*-*1*, which encodes the PELOTA protein conferring resistance to the pepper yellow leaf curl Indonesia virus (PepYLCIV) and the pepper yellow leaf curl Aceh virus (PepYLCAV). This gene was mapped to chromosome 5 using an F_2_ population derived from a cross between a resistant line (BaPep-5) and a susceptible line (BaPep-4). Whole-genome resequencing identified a single nucleotide polymorphism (A to G) at the splice site of the 9th intron in the pelota *CaPelota* gene of BaPep-5. This mutation resulted in the inclusion of the 9th intron in the transcript, adding 28 amino acids to the *Ca* PELOTA protein without inducing a frameshift mutation. The findings of this study were also supported by (Prins et al. 2019), who reported that the artificial Macor (*C*. *annuum*), having mutant allele of *CaPelo*, possesses a deletion of three amino acids in the fourth exon, demonstrating resistance against PepYLCIV, the pepper leaf curl virus, and the pepper huasteco yellow vein virus. Moreover, silencing *CaPelo* gene in a susceptible pepper variety (No.218) through a virus-induced gene silencing (VIGS) approach conferred resistance to PepYLCIV, mirroring the resistant phenotype observed in BaPep-5. More recently, it was demonstrated that *pepy-1* is a leaky allele, instead of complete loss-of-function, that reduces the activity of PELOTA as a begomovirus susceptibility factor, enhancing virus resistance without severely compromising plant growth and development (Onouchi et al. 2025). This fine balance may explain why the leaky allele was naturally selected in pepper and is useful in resistance breeding.

### Knockdown and knockout of *Pelo *expression in begomovirus-susceptible plants converts them to a begomovirus-resistant host

The study by Ren et al. (2022) reconfirmed the role of *Pelo* gene as a viral susceptibility factor, as knockdown of *Pelo* expression in susceptible *Nicotiana benthamiana* rendered it resistant against the same group of begomoviruses. It also showed for the first time that, *Pelo* gene not only confers resistance against TYLCV, but against other begomoviruses as well i.e. tomato yellow leaf curl China virus, tomato leaf curl Yunnan virus, and beet curly top virus. Additionally, they also showed that *ty-5* containing tomato line AVTO1227 exhibited effective resistance to both the representative begomoviruses, which was attributed to a significantly reduced accumulation of viral DNA compared to susceptible tomato line Moneymaker, as demonstrated through southern blotting and quantitative polymerase chain reaction (qPCR). Similarly, Pramanik et al. (2021) used the CRISPR/Cas9 system to knock out the *Pelo* gene in the tomato cultivar BN-86. This resulted in the generation of *SlPelo* knockout lines carrying biallelic indel mutations and exhibited host-mediated immunity against TYLCV. Pathogen resistance assays in these mutant lines confirmed reduced TYLCV accumulation and restricted movement of the viral transcripts to non-inoculated plant parts. Thus, the mutant *SlPelo* tomato lines were successful in inhibiting the TYLCV accumulation.

## Melon *Pelo* acts as a susceptibility factor to tomato yellow leaf curl virus

*Pelo* gene functions as a susceptibility factor not only in tomatoes and other solanaceous crops, but also in cucurbitaceous crops, such as melon. Siskos et al. (2023) explored whether wild-type allele of the melon *Pelo* gene is an anchor for begomovirus pathogenicity and whether mutations in the wild-type allele could confer resistance to begomoviruses. They demonstrated that complementing the wild-type *Pelota* allele into a *ty-5* containing tomato line (harboring mutant allele *pelota*) restored susceptibility to TYLCV, as confirmed by increased viral titers and symptom development. However, *Pelota* allele from melon did not restore susceptibility to beet curly top virus (BCTV), a *Curtovirus*, in *ty-5* containing tomatoe lines. This indicates that a specific position of *Pelo* might be related to the susceptibility of certain geminiviruses. This study highlighted the widespread role of wild-type *Pelota* allele in begomoviral susceptibility, irrespective of the host families involved.

### PELOTA protein and HBS1 function as virus resistance factor, as SUMOylated PELOTA along with HBS1 confers resistance against turnip mosaic virus

Ge et al. (2023) reported a previously unexplored aspect of PELOTA protein in the dynamics between hosts and RNA viruses. Notably, when *Arabidopsis thaliana* PELOTA (*AtPelota*) and *A*. *thaliana* HBS1 (*AtHBS1*) were co-expressed in *N. benthamiana* infected with turnip mosaic virus (TuMV), there was a significant reduction in both viral RNA and protein accumulation compared to control plants. Additionally, *Arabidopsis* mutants lacking functional PELOTA and HBS1 exhibited greater susceptibility to TuMV infection compared to control plants. Similarly, *N. benthamiana* with silenced *NbPelo* and *NbHBS1* showed increased levels of viral RNA and protein accumulation. These results contrasted with earlier observations, where mutations in the wild-type *Pelota* allele were associated with decreased viral activity (against geminiviruses), and was identified as a factor contributing to viral susceptibility.

**Fig. 4:**
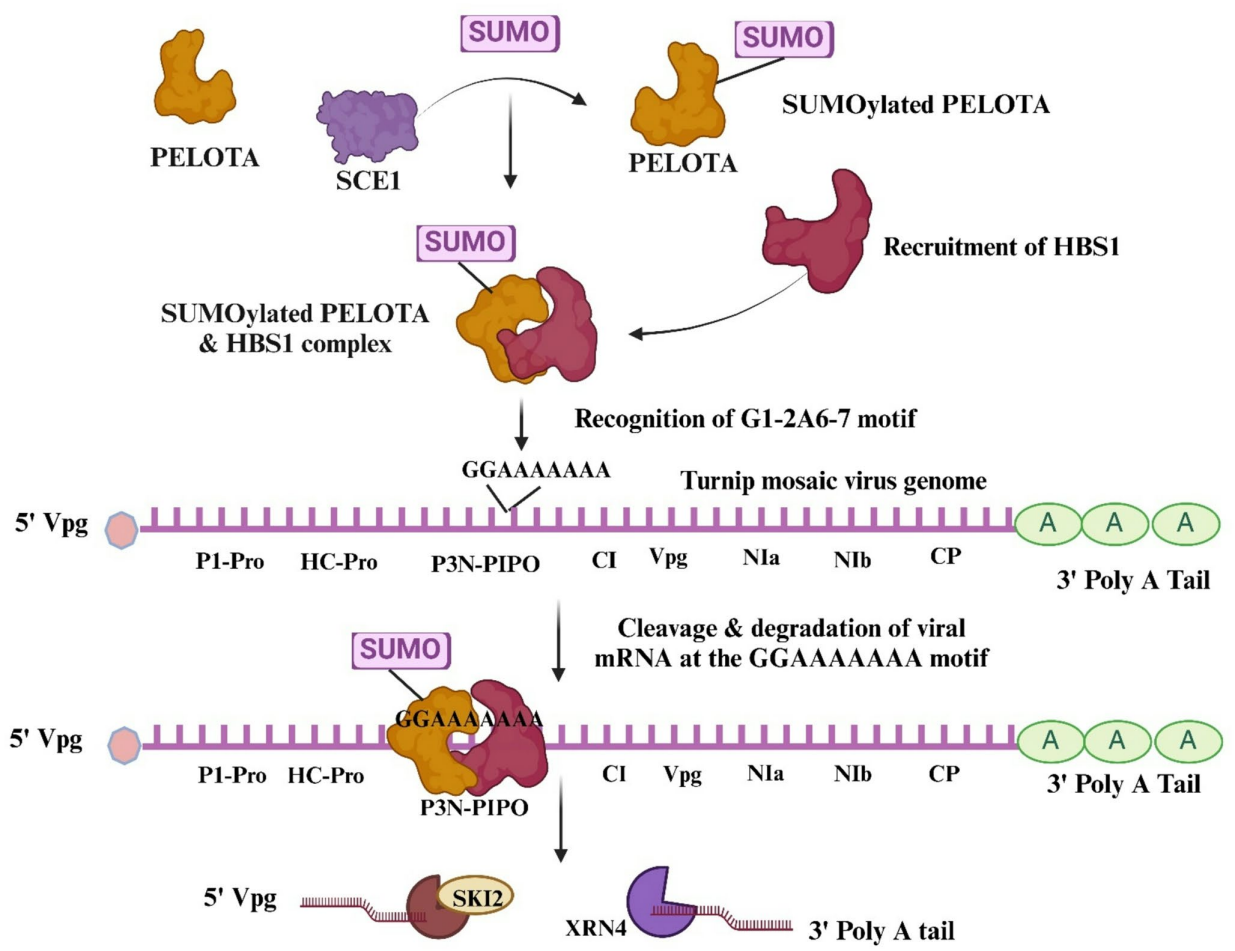
PELOTA-HBS1 mediated degradation of TuMV mRNA through NGD pathway: SCE1 mediated SUMOylation of PELOTA protein facilitates its interaction with the HBS1. This interaction is necessary for the PELOTA-HBS1 complex to identify the conserved G_1−2_A_6−7_ motif in the *P3* cistron of TuMV and cause degradation of viral mRNA. This Figure was created using Biorender.

Additionally, they reported that the accumulation TuMV, a *Potyvirus*, is negatively regulated by PELOTA and HBS1 protein complex, two factors involved in NSD and NGD pathways. For the first time, they demonstrated that SUMOylated PELOTA associated with HBS1 as a complex, can restrict TuMV infection in *A*. *thaliana* (Fig. 4). In TuMV, they identified the *P3-6K1* region of the viral genome as a critical factor for PELOTA-HBS1-mediated resistance. Within this region, PELOTA protein recognizes a conserved G_1−2_A_6−7_ (GGAAAAAA) motif in the *P3* cistron, which is essential for inducing transcriptional slippage and producing the functional movement protein P3N-PIPO (PIPO= Pretty Interesting *Potyviridae* ORF) (Olspert et al. 2015). Additionally, they discovered that SUMOylation of PELOTA protein by the small ubiquitin-related modifier (SUMO) conjugating enzyme 1 (SCE1) is crucial for its interaction with HBS1. This study was the first to identify the PELOTA/HBS1 protein complex as a inhibiting factor for virus accumulation in plants and provided evidence of the importance of SUMOylation in the formation and activity of the PELOTA/HBS1 protein complex.

### Turnip mosaic virus *NIb* decoys PELOTA SUMOylation and promotes virus accumulation

**Fig. 5:**
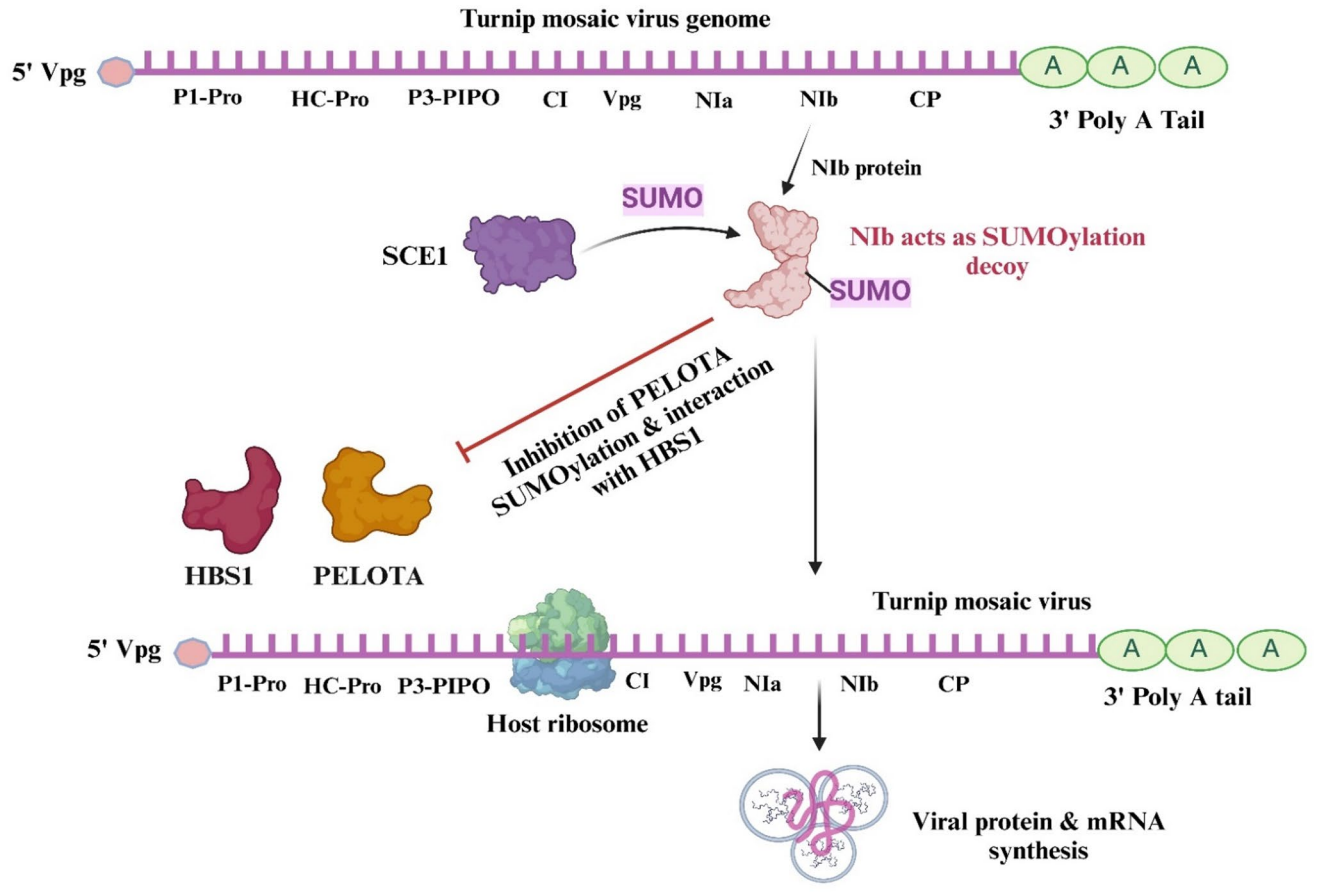
Inhibition of PELOTA SUMOylation by TuMV *NIb protein*: TuMV *NIb* protein acts as a SUMOylation decoy, competing with the SCE1 for PELOTA SUMOylation. By reducing PELOTA SUMOylation, *NIb* disrupts the interaction between PELOTA protein and HBS1, and prevents the formation of PELOTA-HBS1 complex required to degrade viral mRNA. This figure was created using Biorender.

Recently, Ge et al. (2025) reported on how the turnip mosaic virus (TuMV) disrupts the function of PELOTA protein-mediated RNA quality control to facilitate its infection. This study further emphasizes the role of the PELOTA protein as a viral susceptibility factor under specific conditions. They found that TuMV NIb protein encoded by *NIb* gene acts as a SUMOylation decoy, competing with the host SCE1 for PELOTA SUMOylation (Fig. 5). SUMOylation is a post-translational modification involving the attachment of small SUMO proteins to target proteins, which typically alters the stability and function of those proteins (Sabarit and Bejarano 2024). By reducing the SUMOylation of the PELOTA protein, NIb protein inhibits PELOTA's interaction with HBS1, effectively preventing the formation of the PELOTA-HBS1 complex leading to the stability of the viral mRNA. This mechanism allows the virus to replicate and spread efficiently within the host plant. The findings underscore the intricate interplay between plant defensive mechanisms and viral counter-strategies, illustrating how viruses can manipulate host biological processes to enhance infection.

## The paradox of PELOTA: negative impact on plant health if targeted for pathogen resistance

As discussed in the beginning, PELOTA is known primarily for its involvement in ribosome rescue and mRNA surveillance. However, it has a multifaceted role in plant growth and development. When PELOTA was targeted for developing resistance against a particular pathogen, various negative impacts on the host physiology have been observed. Ding et al. (2018) reported that an ethyl methane sulfonate (EMS)-derived mutant of *Oryza sativa Pelo*-like gene (*OsPelo*) exhibited a defective root system and leaf spotting during the early stage of development (while conferring resistance to rice bacterial blight pathogen *Xanthomonas oryzae* pv. *oryzae*). Moreover, the mutation of *OsPelo* resulted in the downregulation of genes involved in the translation, metabolic process, transport, and photosynthetic pathways Similarly, in *Arabidopsis thaliana*, PELOTA protein contributes to germination efficiency and post-embryonic morphogenesis by preventing aberrant co-translational decay of key developmental mRNAs. It was found that null mutants of *Pelo* and *HBS1* displayed significantly reduced germination rates compared to wild type plants, suggesting the important role of PELOTA protein in early developmental stages (Guo and Gregory 2023). Clearly, these findings suggest a context-dependent modulation of PELOTA’s activity, where the spectrum of mRNAs regulated by PELOTA protein may vary between standard growth conditions and those associated with immune activation. Such a framework harmonizes the fundamental housekeeping functions of PELOTA with the phenotypic manifestations observed in loss-of-function mutants, which frequently exhibit developmental defects due to the disruption of general translational accuracy. Concurrently, the reprogramming induced by pathogens may direct PELOTA’s activity towards a more specialized array of transcripts linked to defensive mechanisms. Therefore, elucidating the balance between PELOTA’s foundational role and the specificity of its targets under biotic stress is essential. This understanding also supports the potential for harnessing PELOTA-mediated pathways in resistance engineering with minimal impact on plant growth and development.

## Conclusions and future perspectives

Throughout this article, the discussion on the mRNA surveillance factor PELOTA has revealed its critical role in RNA quality control, virus susceptibility, and/or resistance, and plant growth and development. As stated above, *Pelo* gene in its wild-type and/or mutant form exhibits a unique role in plant-virus interaction, functioning as a susceptibility factor for begomoviruses while acting as a resistance factor against potyviruses. This contrasting behaviour underscores the unique nature of PELOTA protein, operating either as a facilitator or an inhibitor of viral infection. Despite the notable advancements made in understanding how the PELOTA protein functions as an mRNA surveillance factor, much remains unclear regarding its role in influencing susceptibility or resistance to plant viruses under certain conditions. So, unravelling the intricate dynamics of host-virus interactions may provide more clarity on this host-virus arms race.

Moreover, mutational disruption of the wild-type allele of *Pelo* gene through knockout or knockdown strategies is likely to result in aberrant mRNA accumulation, thereby promoting the generation of transposable elements and/or defective proteins that can compromise multiple physiological processes in plants. Thus, targeting *Pelo* gene to develop pathogen resistance in plants might cause a severe negative impact on plant growth and development, which raises a serious concern about targeting it through a knockout and/or knockdown-mediated approach to develop virus-resistant plants. Paradoxically, the mutant/degraded PELOTA protein may not help in viral protein synthesis, which could lead to virus resistance in plants. Thus, a key future challenge is to decipher the mechanistic basis that determines whether the PELOTA protein behaves as a “friend” or “foe” to the plants under certain pathosystems. It is possible that virus-specific motifs within the genomic components of certain viruses (e.g. begomoviruses) modulate how PELOTA engages host translational machinery. Alternatively, other host factors involved in the mRNA surveillance activity may govern whether PELOTA facilitates viral replication or inhibits it. Advanced omics-based techniques, such as comparative interactomics, domain-swapping of viruses, will provide a critical understanding of these aspects. Additionally, specific immune-related or virus-derived transcripts may be preferentially recognized by PELOTA during viral infection. Employing ribosome profiling alongside transcriptomics-based studies in *Pelo* mutants could provide insights into transcript-level specificity and the underlying mechanisms involved.

From an applied standpoint, future crop engineering strategies should aim to exploit PELOTA’s pathogen-specific roles while minimizing its negative effect on plant growth and development. Approaches such as allele-specific editing, engineered tissue-specific suppression of PELOTA, may be useful in conferring viral resistance. Moreover, identifying amino acid substitutions that confer resistance against begomoviruses, or those required to retain resistance against potyviruses, while having no negative effects on normal plant development, would be ideal. Such strategies could ultimately transform PELOTA from a paradoxical target into a ‘tuneable’ target for durable viral resistance without compromising plant growth and development.

## Data Availability

No datasets were generated or analysed during the current study.

## Abbreviations

RQC: RNA quality control
TYLCV: Tomato yellow lLeaf curl virus
TYLCD: Tomato yellow leaf curl disease
NMD: Nonsense-mediated mRNA decay
NSD: Nonstop decay
NGD: No-go decay
PTC: Premature termination codon
UTR: Untranslated region
PVX: Potato virus X
TCV: Turnip crinkle virus
iTCs: internally located termination codon
RaQC: Ribosome associated quality control
HBS1: Hsp70 subfamily B suppressor 1
eRF1: Eukaryotic release factor 1
GTP: Guanosine triphosphate
ABCE1: ATP-binding cassette sub-family E member 1
SKI: Super killer
XRN1: Exoribonuclease 1
SRBSDV: Southern rice black-streaked dwarf virus
WBPH: White-backed plant hopper
PepYLCIV: Pepper yellow leaf curl Indonesia virus
PepYLCAV: Pepper yellow leaf curl Aceh virus
BCTV: Beet curly top virus
TuMV: Turnip mosaic virus
PIPO: Pretty interesting *Potyviridae* ORF
SUMO: Small ubiquitin-related modifier
SCE1: SUMO conjugating enzyme 1
